# Optimal processing of tongue swab samples for *Mycobacterium tuberculosis* detection by the Xpert MTB/RIF Ultra assay

**DOI:** 10.1128/spectrum.02403-24

**Published:** 2025-01-28

**Authors:** Gayatri Shankar Chilambi, Robert Reiss, Naranjargal Daivaa, Padmapriya Banada, Margaretha De Vos, Adam Penn-Nicholson, David Alland

**Affiliations:** 1 Department of Medicine, Rutgers New Jersey Medical School, Newark, New Jersey, USA; 2 FIND 91635 , Geneva, Switzerland; Johns Hopkins University, Baltimore, Maryland, USA

**Keywords:** molecular diagnostics, tongue swab, tuberculosis, Xpert Ultra

## Abstract

Tongue swabs represent a potential alternative to sputum as a sample type for detecting pulmonary tuberculosis (TB) with molecular diagnostic tests. The methods used to process tongue swabs for testing in the World Health Organization-recommended Xpert MTB/RIF Ultra (Xpert Ultra) assay vary greatly. Here, we aimed to identify the optimal tongue swab processing for Xpert Ultra testing. We compared four methods for treating dry tongue swabs with the Xpert Sample Reagent (SR) mixed with various concentrations of Tris–EDTA–Tween to treatment with SR alone or to a commonly used heat inactivation protocol. In each condition, swabs obtained from volunteers without TB were placed into test buffer spiked with known amounts of *Mycobacterium tuberculosis* (*Mtb*) H37Rv-mc^2^6230. Swabs processed with 1:1 diluted SR buffer had the lowest *Mtb* limit of detection (LOD) at 22.7 CFU/700 µL (95% confidence interval (CI) 14.2–31.2), followed by 2:1 diluted SR buffer at 30.3 CFU/700 µL (95% CI 19.9–40.7), neat SR at 30.9 CFU/700 µL (95% CI 21.5–40.3), and SR prefilled in the Xpert Ultra at 57.1 CFU/700 µL (95% CI 42.4–71.7). Swabs processed using the heat-based protocol had the highest LOD (77.6 CFU/700 µL; 95% CI 51.2–104.0). Similar findings were observed for the LOD of RIF susceptibility. The 2:1 diluted SR buffer condition produced similar LODs when swabs were tested in the presence of sputum matrix or phosphate buffer saline. Further studies are needed to assess the performance of this processing protocol in a clinical setting.

**IMPORTANCE:**

Xpert MTB/RIF Ultra (Xpert Ultra) is approved by the World Health Organization for the diagnosis of tuberculosis (TB). This test is typically performed using sputum specimens obtained from people with presumptive TB. In order to inactivate *Mycobacterium tuberculosis* (*Mtb*) and aid liquefaction, sputum must be mixed with Xpert SR prior to transfer into the Xpert Ultra. However, some people under evaluation for TB are unable to produce sputum. Alternative sample types for TB diagnosis would, therefore, be of value. Oral swabs, including tongue swabs, have shown promise, but there are technical challenges associated with sample processing. In this study, several new tongue swab processing conditions were evaluated by utilizing SR, either neat or diluted in buffer. The ability of Xpert Ultra to detect TB was improved under these conditions compared with the previously published heat-processing method; processing steps were simplified; and technical challenges were overcome.

## INTRODUCTION

Tuberculosis (TB) remains a major public health threat. Globally, only 7.5 million people were newly diagnosed with TB in 2022 (1), while an estimated 10.6 million actually developed TB during that year, leaving 3.1 million people undiagnosed or underreported (1). Delays in diagnosis contribute to low TB treatment rates and consequently to increased mortality and disease transmission (1, 2).

Most existing TB diagnostics rely on sputum specimens for *Mycobacterium tuberculosis* (*Mtb*) complex detection (2). These include sputum-smear microscopy, culture-based tests, or molecular World Health Organization-recommended diagnostic tests, such as Xpert MTB/RIF and Xpert MTB/RIF Ultra (Xpert Ultra; Cepheid, Inc., Sunnyvale, CA), the Truenat assays MTB, MTB Plus, and MTB RIF-Dx (Molbio Diagnostics, Ltd., Verna, Goa, India), moderate complexity assays, such as the Abbott RealTi*me* MTB assays (Abbott Laboratories, Abbott Park, USA), Roche Cobas MTB (Hoffmann-La Roche, Basel, Switzerland), Fluorotype MTBDR (Bruker/Hain Lifescience, Nehren, Germany), BD MAX MDR-TB (Becton, Dickinson and Company, Franklin Lakes, USA), and the TB-LAMP assay (Eiken Chemical, Tokyo, Japan) (2). However, TB diagnosis with sputum-based technologies is associated with challenges, including the inability of some patients to produce sputum, such as younger children or people living with HIV (3–7). Several non-sputum-based diagnostics are under investigation to address these needs, utilizing specimen types, such as blood, stool, urine, and breath aerosols.

Considerable effort has been undertaken to study the potential of tongue swabs as an alternative non-invasive oral sample type for pulmonary TB (8, 9). Previous studies with tongue swabs have reported 52–91% sensitivity in adults, with considerable variation according to sample processing methods, sampling site, swab type, storage conditions, and analysis approaches (9). These studies evaluated the ability of automated TB detection platforms, such as Xpert Ultra (8, 10–13) and MTB Ultima (Molbio Diagnostics, Ltd., Verna, Goa, India) (10), or in-house PCR tests (14), to detect *Mtb* in tongue swabs either spiked with *Mtb* bacilli or confirmed TB-positive by a reference standard.

Xpert Ultra is known to have a low limit of detection (LOD) and has optimal analytical sensitivity to detect *Mtb* in non-sputum specimens, such as cerebrospinal fluid (CSF), gastric aspirates, and tissue biopsies, using processing protocols based on the Cepheid proprietary Xpert sample reagent (SR) (2, 15–18). Several studies assessed TB detection when tongue swabs were processed with SR based on the existing sputum-based Xpert MTB/RIF Ultra (Ultra) protocol (8, 11). Andama et al. showed analytically with spiked tongue swabs that a single tongue swab treated with SR yielded a higher LOD in comparison to processing double swab with SR or single swab with a heat-based processing approach. However, isolated reports of over-pressurization errors (19) were observed with Xpert Ultra testing of tongue swab samples based on current heat-based sample processing protocols. Developing an SR-based tongue swab processing protocol optimized for Xpert Ultra testing that does not result in over-pressure errors and enables improved assay sensitivity would be helpful.

The objective of this study was to evaluate the impact of four modifications of tongue swab sample processing conditions with different dilutions of Xpert SR on the performance of Xpert Ultra for detection of *Mtb* and rifampicin (RIF) resistance when compared with the existing heat-based inactivation protocol (8) for the Xpert Ultra assay. The impact of tongue biomass, sputum matrix swab, and buffer on performance using one selected sample processing condition was also evaluated.

## MATERIALS AND METHODS

### Study participants and swab sample collection

Healthy volunteers (aged >18 years) were recruited for swab donation using flyers posted in the International Center for Public Health building at Rutgers. The swabs were collected by trained investigators according to a standard protocol but were not examined pathologically. Participants were asked not to eat or drink, use tobacco or oral hygiene products, or expectorate sputum up to 30 min before collecting their tongue swabs. Participants were provided with sterile, single-use, and individually packaged Copan FLOQSwabs (520CS01) to self-collect, rolling the swab head along the breadth of their mid-tongue or back half of the dorsum of the tongue without touching their teeth or throat, in exactly 10 s. After using a single FLOQSwab, the swab head was snapped at the 30 mm breakpoint into a 2 mL sterile and empty screw cap collection tube. The closed tubes were placed inside a cooler box in a leak-proof biohazard bag. All the swabs were stored at 2–8°C for less than a week before testing.

In order to evaluate the impact of sputum matrix swabs on the Xpert Ultra performance, sputum from leftover clinical specimens collected from the University Hospital Microbiology Lab (University Hospital, Newark, NJ, USA) was used.

For tongue swab collection, informed consent (paper/electronic email or internet/oral script) document was provided to all research subjects for the tongue swab collection under IRB protocol #Pro2021002454. Sputum was collected under a waiver of consent under IRB protocols 020160000657 and 0120090104 from deidentified clinical samples submitted to the University Hospital’s microbiology laboratory, which normally would have been discarded after clinical testing for a non-tuberculosis suspected illness.

### Preparation and quantitation of concentrated viable *Mtb* cell stock

Titered stocks of an attenuated version of the *Mtb* H37Rv (H37Rv-mc^2^6230) laboratory strain were prepared and quantified, as previously described (17). The *Mtb* cells were inoculated in a ratio of 1:100 in 10 mL of 7H9 broth supplemented with 10% Middlebrook OADC growth supplement, 0.05% Tween 80 (Sigma Aldrich, St. Louis, MO), and 24 µg/mL of calcium pantheonate (Sigma Aldrich, St. Louis, MO). *Mtb* grown to an optical density at 600 nm (OD600) of 0.6–0.8 was sub-cultured twice before dilutions were prepared to quantify colony-forming units (CFU); the culture was then divided into 500 µL aliquots and stored at −80°C until use. The 10^−5^, 10^−6^, and 10^−7^ fold-dilutions were plated in triplicate on 7H10 agar plates supplemented with 10% Middlebrook OADC growth supplement and 24 µg/mL calcium pantheonate (17). Plates were incubated for 2 to 3 weeks at 37°C, and the CFU/mL of the culture stock was estimated from plates with colony counts ranging between 300 and 10.

### Cell preparation and dilution for spiking

Cell dilutions were prepared, as previously described (17). The quantified stocks of *Mtb* were removed from −80°C storage and thawed on ice. Thereafter, 500 µL of supplemented 7H9 media was added to a 500 µL aliquot of the quantified *Mtb* stock, which was mixed by vortexing for 30 s at medium speed and placed on ice for 6 min to allow aerosolized particles to settle. The mixture was then sonicated, and serial dilutions between 10 and 10^7^ CFU/mL were made with supplemented 7H9. The stock suspensions were stored at 4°C and sonicated once for 30 s on the subsequent day. Stock suspension used on additional days (days 3–7) was vortexed at medium speed for 2 min and restricted to a maximum of one such vortex every 24 h. For any given comparison, all cell concentrations were prepared from the same aliquot of quantified stock.

### Comparing a heat-based swab processing protocol to a modified Xpert Ultra sputum testing-based protocol

The performance of a modified Xpert Ultra sputum testing-based protocol versus heat-based inactivation was assessed using human volunteer tongue swab samples placed in 700 µL of the Tris–EDTA–Tween (TET) buffer (50 mM Tris, 0.1 mM EDTA, 0.1% Tween20, pH 8.4) containing 0, 7, or 14 CFU of *Mtb*. In this study, all experiments have been presented as CFU per 700 µL to account for the actual cell numbers used per volume of buffer and not 1 mL to avoid any unknown differences in cell numbers since (a) only 500 µL of the sample is transferred to the Xpert Ultra cartridge for TB testing, and (b) a small volume of the buffer might be absorbed by the swab itself. In the heat-based inactivation protocol, 700 µL of the TET buffer spiked with *Mtb* was transferred by a pipette to the tube containing the swab head and heated for 10 min at 95°C. Samples were cooled to room temperature before vortexing for 15 s and pulse centrifugation. Subsequently, 500 µL of the supernatant was transferred into the Xpert Ultra cartridge sample chamber prefilled with 1.5 mL of TET buffer. In the modified Xpert Ultra sputum testing-based protocol, an SR–TET buffer was prepared by mixing SR and TET buffer in a 2:1 ratio; 700 µL of this buffer aliquot was spiked with various CFUs of *Mtb* and transferred to a tube containing a swab head. Then, 500 µL from this swab-containing tube was transferred to the Xpert Ultra cartridge sample chamber prefilled with 1.5 mL of the SR:TET (2:1) buffer and incubated at room temperature for 15 min.

### Comparing a heat-based swab processing protocol to four different SR-based swab processing protocols that did not include a heating step

**Fig 1:**
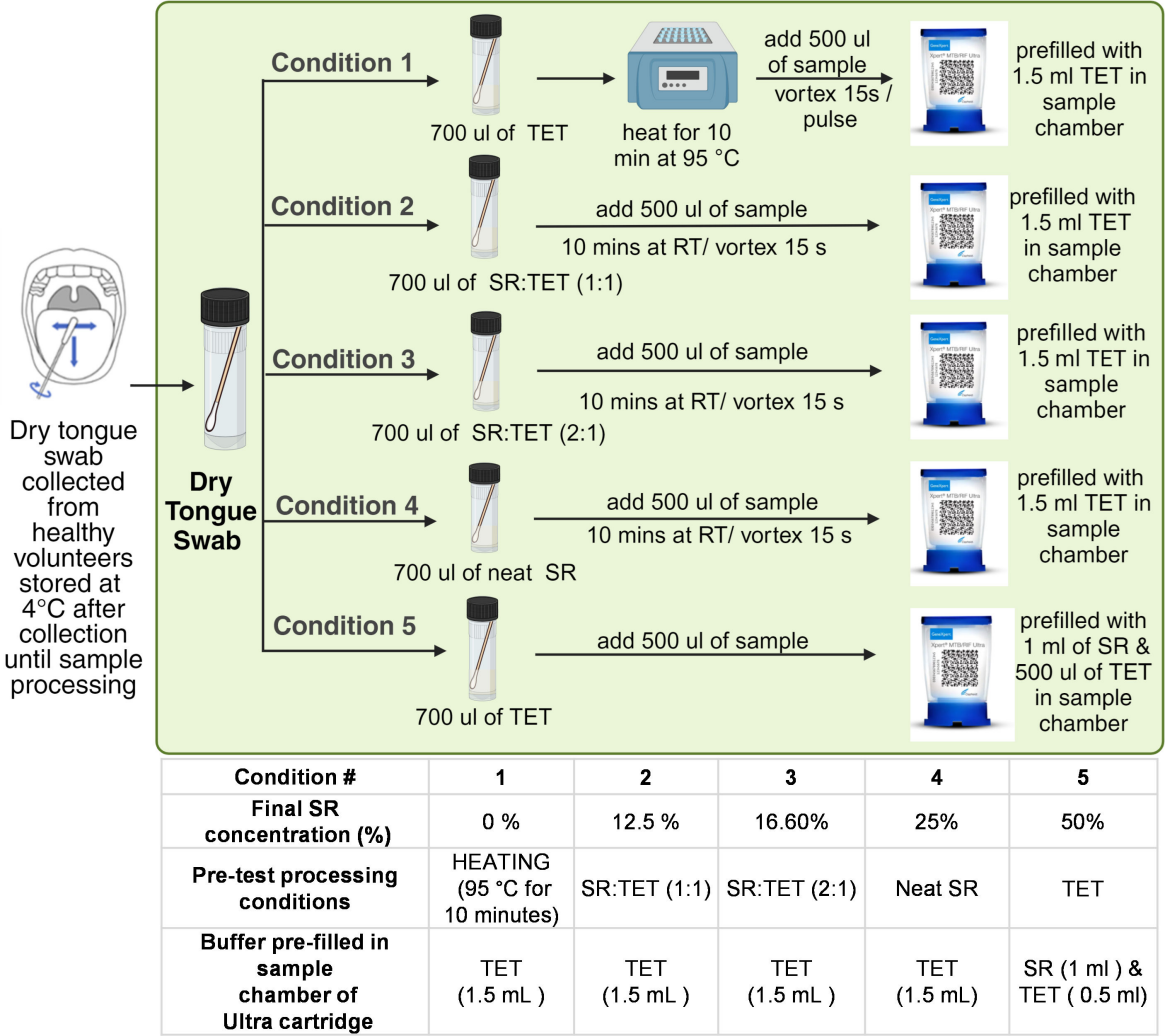
Sample processing workflow showing how tongue swabs (spiked or unspiked) were processed before loading into the Xpert MTB/RIF Ultra cartridge. RT, room temperature; SR, Xpert sample reagent; TET, Tris–EDTA–Tween buffer. Created with BioRender.com.

Tongue swabs from human volunteers were placed in 700 µL of TET buffer either spiked or unspiked with various concentrations of *Mtb* CFUs and thereafter processed using five different sample processing conditions, referred to as conditions 1–5 (Fig. 1). Each of these protocols tested included either TET or a combination of TET buffer and SR as the sample processing reagent. Each sample processing condition was tested simultaneously, with final concentrations of *Mtb* of 0, 7, 14, 28, and 56 CFU/700 µL. Conditions 1 and 5 were tested at two additional concentrations of 112 and 336 CFU/700 µL, respectively.

Condition 1 was the existing heat-based inactivation protocol for the Xpert Ultra assay and used as a comparator in this study. In sample processing conditions 2, 3, and 4, 700 µL of SR and TET buffer was prepared in a 1:1 or 2:1 ratio or neat SR, respectively, with the 2:1 ratio representing the SR:sample proportions used for treating sputum samples with Ultra. These 700 µL buffer aliquots were then spiked with *Mtb* and transferred to the tube containing the swab head, vortexed briefly, and incubated at room temperature. After 10 min of incubation, 500 µL of the sample was transferred to the Xpert Ultra cartridge sample chamber prefilled with 1.5 mL of TET buffer. Finally, in condition 5, 700 µL of TET buffer spiked with *Mtb* was added to the tube containing the swab, and 500 µL of the sample from the tube was loaded into the Xpert Ultra cartridge prefilled with 1 mL of SR and 500 µL of TET buffer. Condition 5 was designed to test the assay performance for tongue swab heads that were exposed briefly to higher final concentrations of SR when compared to conditions 2–4.

### Xpert Ultra performance assessment

Xpert Ultra performance under each sample processing condition was assessed according to the *Mtb* dilution until *Mtb* positivity or RIF resistance could be detected in 100% of samples. Any test that resulted in an invalid or error output from the GeneXpert instrument was excluded from the primary analysis but recorded for error reporting. The LOD under each condition was also calculated and defined as the lowest concentration of the analyte (IS*6110*/IS*1081* for *Mtb* positivity and *rpoB* for RIF susceptibility) detected with 95% certainty (17). Mean IS*6110*/IS*1081* and *rpoB* cycle threshold (Ct) values were measured for each condition.

One sample processing condition was then selected based on the associated LOD and biosafety considerations for further analyses, which included the impact of tongue biomass, sputum matrix swab, and use of phosphate-buffered saline (PBS) instead of Tris–EDTA–Tween, on the Xpert Ultra performance under the selected condition. In-cartridge pressure values of Xpert Ultra tests performed with tongue and sputum matrix swabs were noted. A modified version of our protocol with SR and phosphate buffer is also available for validation in resource-limited and high-burden TB settings (20).

### Statistical analysis

All statistical analyses were performed using R Statistical Software version 4.3.1 (R Foundation for Statistical Computing, Vienna, Austria) or GraphPad Prism Version 9.5.1 (733).

For comparing the heat-based swab processing protocol to the modified Xpert Ultra sputum testing-based protocol, the common odds ratio for the association between the sample processing condition and the *Mtb/rpoB* detection over three levels of cell concentration was calculated and tested using the Cochran–Mantel–Haenszel Chi-squared test. Fisher’s exact test was used to test the stratum-specific associations. Probit regression was used to model the detection rate as a function of concentration, and LODs were calculated using inverse estimation. The LOD estimates for two conditions were considered significantly different if there was no overlap between the Wald 95% confidence intervals for each condition.

For comparing the heat-based swab processing protocol to the four different SR-based swab processing protocols that did not include a heating step, two-way analysis of variance was used to examine the differences in Ct values between concentrations under multiple sample processing conditions. The Ct values of conditions 1 and 5 were compared using independent sample *t*-tests at concentrations greater than 56 CFU/700 µL. Only the Ct values of samples positive for *Mtb* (in the case of IS*6110*/IS*1081* Ct) (Fig. 3a; Table S3) and RIF susceptibility (in the case of *rpoB* Ct) (Fig. 3b; Table S4) were considered for statistical analysis. Post-hoc pairwise comparisons were conducted using Tukey’s multiple comparison test.

**Fig 2:**
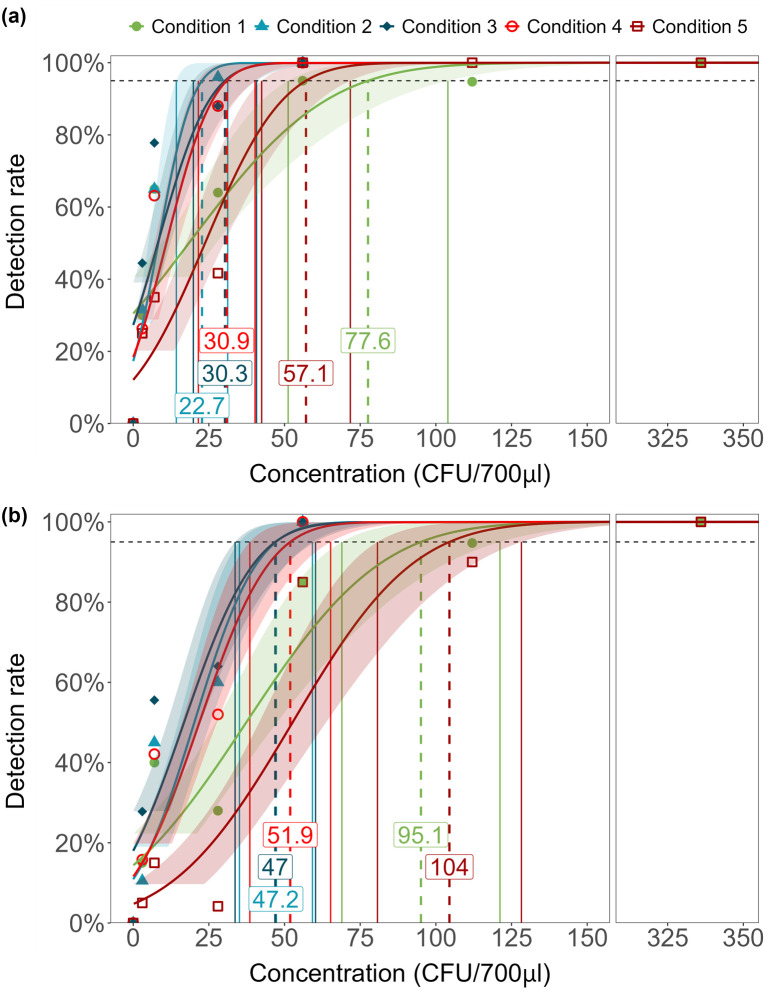
Limit of detection of (a) *Mtb* positivity and (b) RIF susceptibility for tongue swabs under sample processing conditions 1–5. Dashed lines indicate concentration at the 95% limit of detection, and solid lines indicate the upper and lower bounds of Wald 95% CI. The numbers in the boxes are labels for concentration at 95% LOD for each condition. CFU, colony-forming unit.

**Fig 3:**
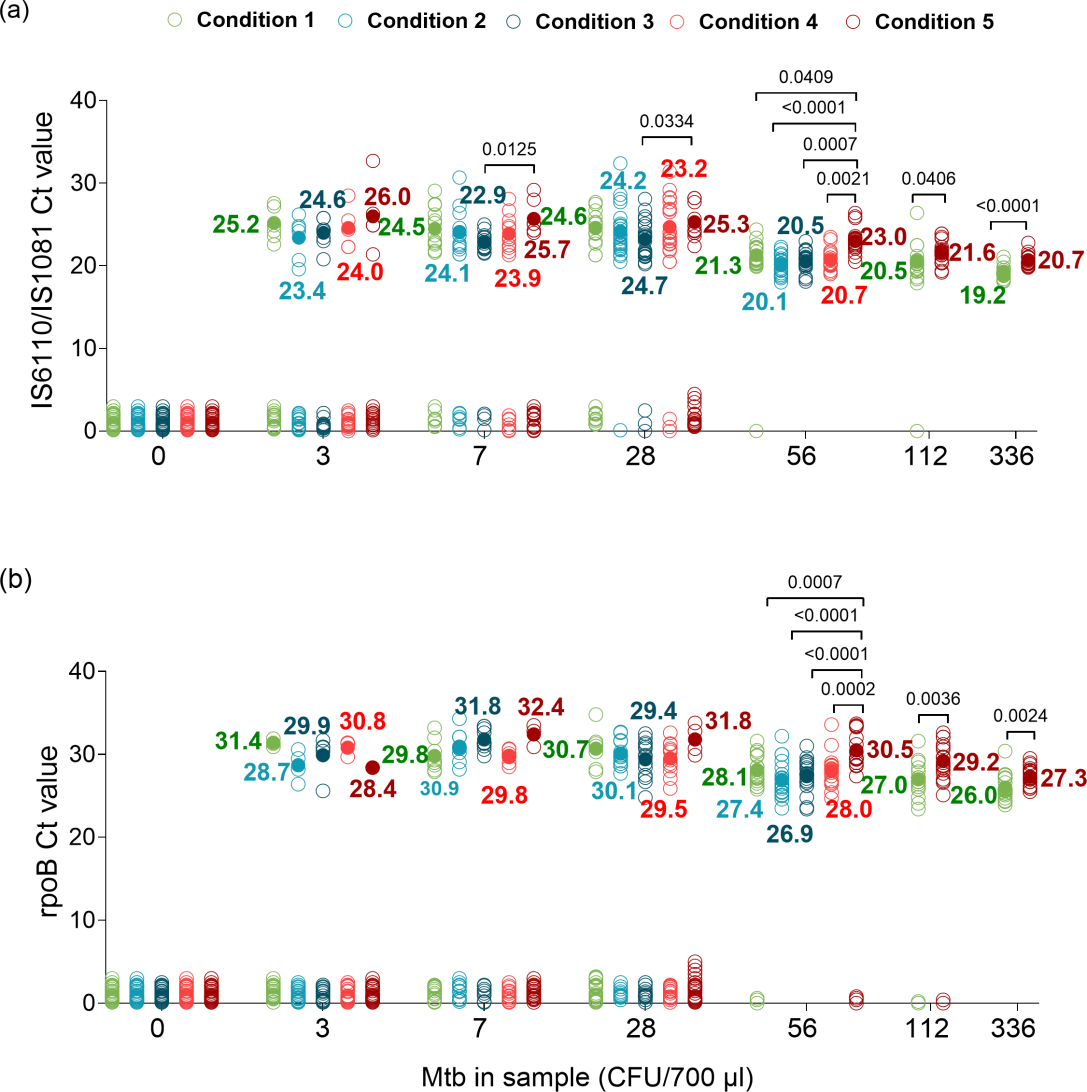
Ct values of (a) IS*6110*/IS*1081* and (b) *rpoB* for tongue swabs under sample processing conditions 1–5. Mean Ct values are indicated by filled circles. CFU, colony-forming unit; Ct, cycle threshold; *Mtb*, *Mycobacterium tuberculosis*.

**Fig 4:**
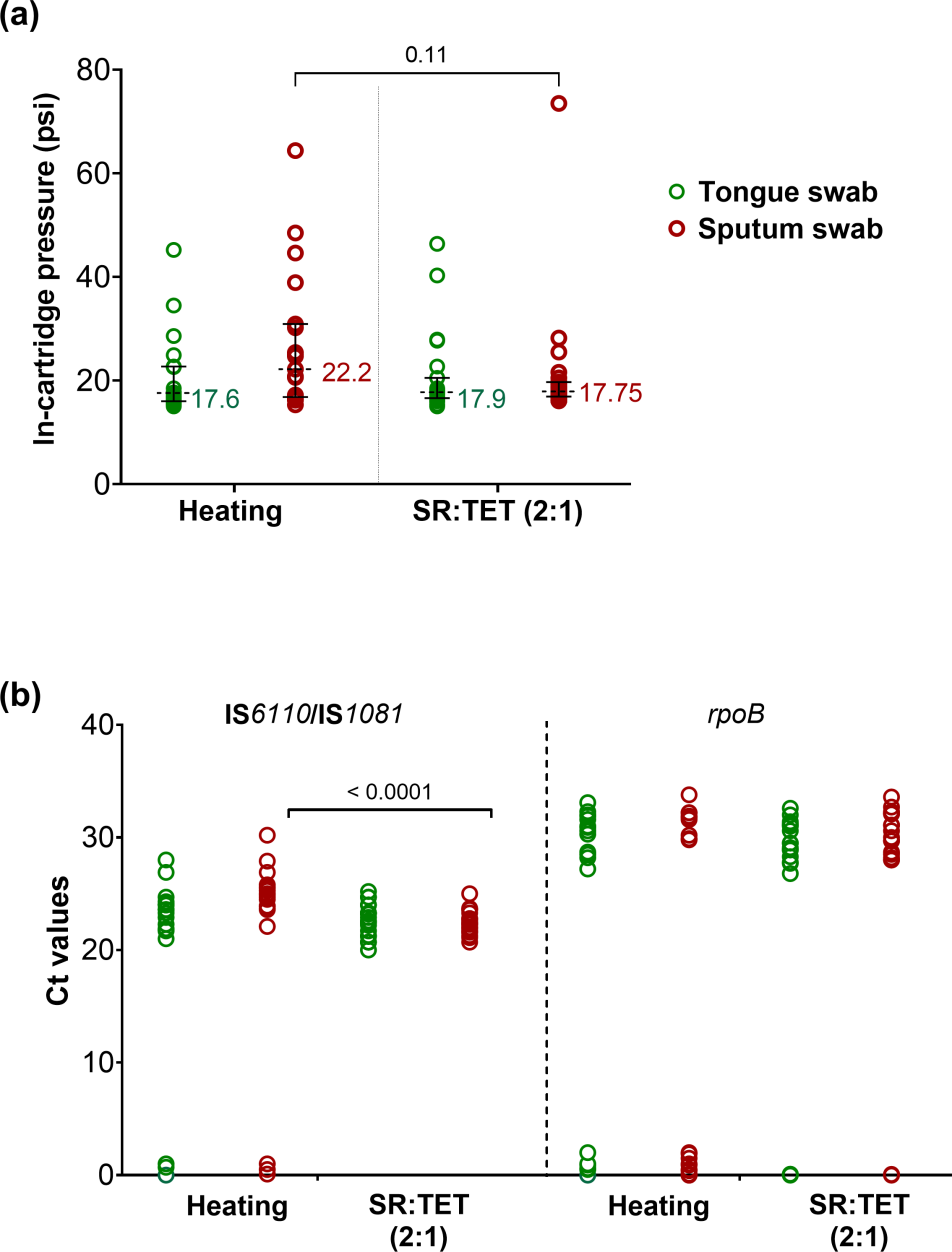
(a) In-cartridge pressure values of tongue and sputum swab samples processed by condition 1 (heat) or 3 (diluted SR: TET 2:1); dotted lines and error bars indicate median and 95% confidence interval, respectively. (b) Xpert Ultra Ct values for tongue swab samples processed by condition 1 (heat) or 3 (diluted SR:TET 2:1) for *IS*6110/*IS*1081 and *rpoB*. Ct values of 0 were included in the graph to represent the negatives but were not used for statistical analysis. CFU, colony-forming unit; Ct, cycle threshold; SR, Xpert sample reagent; TET, Tris–EDTA–Tween buffer. No errors or invalids were observed for this experiment.

**Fig 5:**
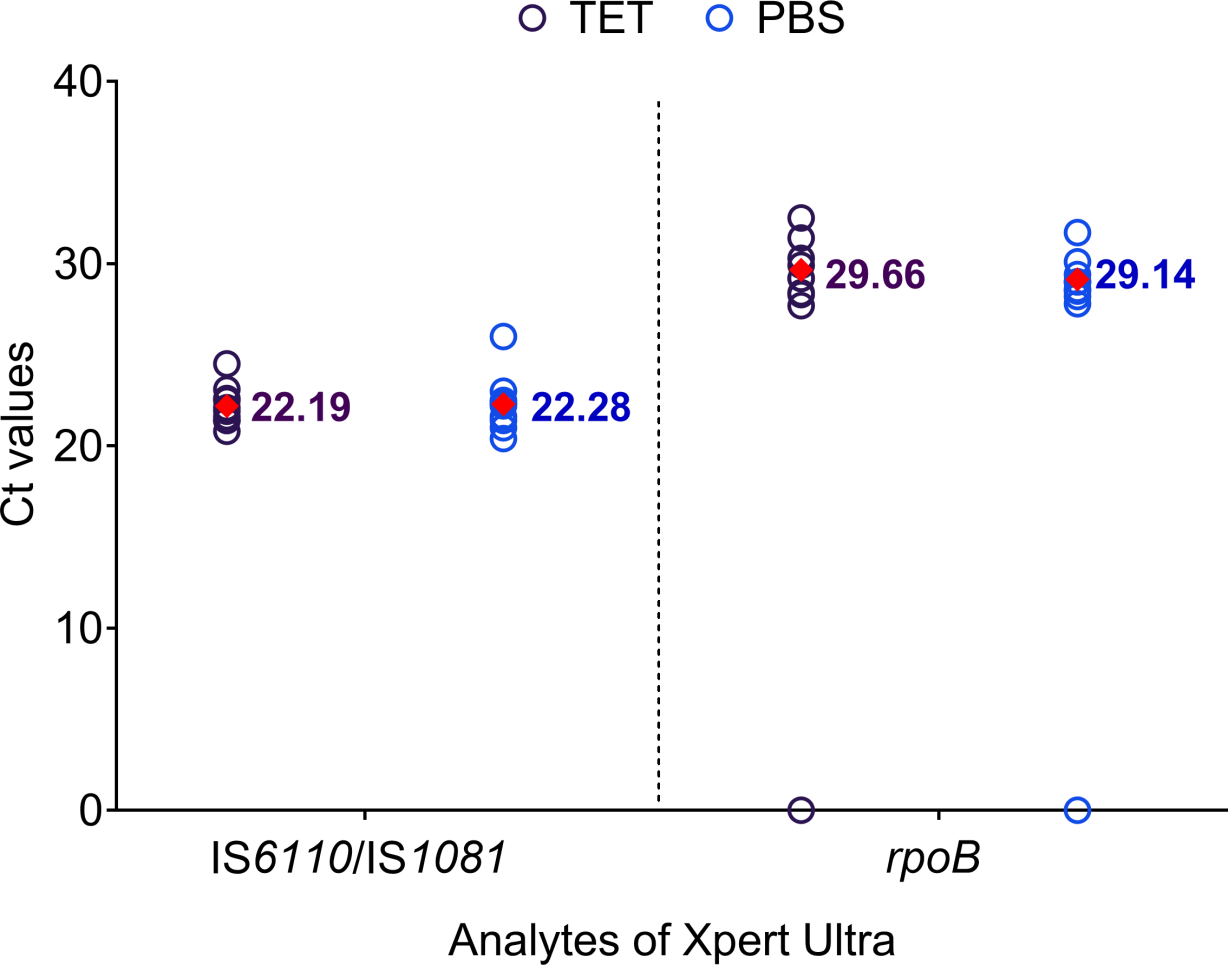
Comparison of IS*6110*/IS*1081* and *rpoB* Ct values at 28 CFU per 700 µL for tongue swab samples processed by condition 3 (diluted SR: TET 2:1) with TET vs PBS as diluent buffer. Mean Ct values are indicated by filled diamonds. Ct values of 0 were included in the graph to represent the negatives but were not used for statistical analysis. Ct, cycle threshold; PBS, phosphate-buffered saline; TET, Tris–EDTA–Tween buffer. No errors or invalids were observed for this experiment.

For conditions 1 and 3, the medians of in-cartridge pressure values were compared using Mood’s median test, and the means of the Ct values were compared using independent sample *t*-tests. Homogeneity of variance for each condition was tested using Levene’s test. Detection rates under differing conditions were compared using Fisher’s exact test. The raw data that were used to create Fig. 2 to 5 are included in Table S1 to S7 available in the supplemental material.

## RESULTS

### Comparing a heat-based swab-processing protocol to a modified Xpert Ultra sputum testing-based protocol

We compared the performances of tongue swabs processed with heat (equivalent to condition 1 below) and a modified sputum testing-based protocol (SBP) where the sample was treated with SR and diluted 2:1 with TET (with a final SR concentration of 66.6%). We tested the common odds ratio of the performance of the SBP and the heat inactivation protocol across three levels of cell concentrations using the Cochran–Mantel–Haenszel Chi-squared test. Xpert Ultra assay results provide information on the presence of *Mtb*, and the presence of RIF susceptibility or resistance based on their respective molecular targets IS*6110*/IS*1081* and *rpoB. Mtb* LOD is usually more sensitive, as observed in cases where IS*6110*/IS*1081* is detected, but *rpoB* remains undetected in the sample (17). The odds of detecting a positive *Mtb* sample were 1.60 times higher in the heat-based inactivation protocol compared to SR treatment (*P* = 0.45), while the odds of detecting RIF susceptibility were 1.83 times higher in the heated prep compared to SBP (*P* = 0.30), although these differences were not statistically significant (Table 1).

**TABLE 1:** Xpert Ultra performance with *Mtb* spiked into buffer with swab samples processed using a heat-based swab-processing protocol versus a modified Xpert Ultra sputum testing-based protocol^*c*^

Analyte		*Mtb* positivity, n/N (%)		*P*-value (Fisher’s exact test)	RIF susceptibility, n/N (%)		*P*-value (Fisher’s exact test)
Condition description		Heating (100% TET)	SBP		Heating (100% TET)	SBP	
Final SR concentration (%)		0%	66.6%		0%	66.6%	
CFU per 700 µL of sample buffer	0	0/4 (0%)	0/4 (0%)	-	0/4 (0%)	0/4 (0%)	-
	7	9/19 (47.4%)	8/20 (40%)	0.7512	4/19 (21.1%)	1/20 (5%)	0.1818
	14	16/16 (100%)	14/15 (93.33%)	0.4839	8/16 (50%)	7/15 (46.67%)	1

				*P*-value (CMH)			*P*-value (CMH)
Common odds ratio (95% CI)		1.60 (0.47–5.44)		0.45	1.83 (0.58–5.79)		0.30
Errors (Total errors/ total tests)		1^*a*^/39	1^*b*^/39		NA	NA	

^*a*^ Error 5007: [rpoB1] probe check failed.

^*b*^ Error 2037: the cartridge integrity test failed at valve position 1390.

^*c*^ CI = confidence interval, CMH = Cochran–Mantel–Haenszel Chi-squared test , SBP = modified Xpert Ultra sputum testing-based protocol, NA and “-” = not applicable.

### Sample processing conditions assessed by limit of detection

We next determined the LOD of samples processed using the five sample processing conditions and then tested with Xpert Ultra assay to estimate the influence of the different tongue swab sample processing conditions on the assay performance. For *Mtb* detection, the LOD (Fig. 2a; Table S1) was lowest for swabs processed by condition 2 at 22.7 CFU/700 µL (95% CI 14.2–31.2), followed by conditions 3 and 4 at 30.3 CFU/700 µL (95% CI 19.9–40.7) and 30.9 CFU/700 µL (95% CI 21.5–40.3), respectively. The LOD of *Mtb* with swabs processed by condition 5 was 57.1 CFU/700 µL (95% CI 42.4–71.7). Swabs processed by condition 1 had the highest LOD of 77.6 CFU/700 µL (95% CI 51.2–104.0).

For detection of RIF susceptibility or resistance, the LOD was based only on whether the output of the Xpert Ultra assay reported RIF as susceptible since no RIF-resistant isolates were tested. Assays that were reported as trace or RIF indeterminant do not indicate a valid RIF result in the Ultra assay and were, therefore, both considered non-results or negative in the RIF LOD calculations (Fig. 2b; Table S2). The LODs of condition 2 (47.2 CFU/700 µL [95% CI 35.1–59.2]), condition 3 (47.0 CFU/700 µL [95% CI, 33.7–60.2]), and condition 4 (51.9 CFU/700 µL [95% CI 38.5–65.2]) were all comparable. A higher LOD of RIF susceptibility was observed in swabs processed by condition 1 (95.1 CFU/700 µL [95% CI 68.9–121.2]) and condition 5 (104.5 CFU/700 µL [95% CI 80.7–128.3]) (Fig. 2b; Table S2). No false positive results for *Mtb* detection or RIF resistance were observed in any of the samples tested.

### Performance of Xpert Ultra by sample processing condition based on cycle threshold values

The IS*6110*/IS*1081* (for *Mtb* detection) and *rpoB* (for RIF susceptibility detection) Ct values resulting from our LOD studies of tongue swab samples processed by conditions 1–5 were plotted based on the numbers of *Mtb* CFU spiked into each sample condition (Fig. 3; Table S3 and S4). A significant difference was observed in the mean IS*6110*/IS*1081* Ct values of conditions 1 and 5 for samples tested at 336 CFU/700 µL (*P* < 0.0001) and 112 CFU/700 µL (*P* = 0.0406). IS*6110*/IS*1081* Ct values of samples tested at 56 CFU/700 µL with condition 5 were significantly different from conditions 1 (*P* = 0.0409), 2 (*P* < 0.0001), 3 (*P* = 0.0007), and 4 (*P* = 0.0021). A significant difference was observed in the mean *rpoB* Ct values in conditions 1 and 5 for samples tested at 336 CFU/700 µL (*P* = 0.0024) and 112 CFU/700 µL (*P* = 0.0036). At 56 CFU/700 µL, a significant difference was observed between the mean *rpoB* Ct values of samples processed by condition 5 versus conditions 1 (*P* = 0.0007), 2 (*P* < 0.0001), 3 (*P* < 0.0001), and 4 (*P* = 0.0002). However, there was no significant difference between *rpoB* Ct values in samples tested at concentrations below 56 CFU/700 µL for any sample processing method.

### Selecting the optimal swab treatment condition

Based on the combined LOD and Ct value findings (Table 2; Fig. 2 and 3; Table S1 to S4) of both the *Mtb* and rifampin resistance components of the assay, conditions 2, 3, and 4 were not statistically different. However, we considered the fact that condition 3 had modestly higher CFU detection rates in samples spiked with lower numbers of CFU (3 and 7 CFU for *Mtb* detection and 3, 7, and 28 CFU for rifampin susceptibility detection, Table 2), along with prior evidence that 2:1 dilutions of SR:sample effectively decontaminate samples (21). These considerations led us to select condition 3 (SR: Tris–EDTA–Tween buffer 2:1) as the optimal tongue swab processing method for further analytical studies. Condition 1 (existing heat-based protocol) continued to be used as a comparator.

**TABLE 2:** Comparative assessment of the Xpert Ultra assay testing of *Mtb* spiked into buffer with tongue swabs by sample processing condition^*f*^^,^^*g*^

Analyte		*Mtb* positivity, n/N (%)					RIF susceptibility, n/N (%)				
Condition		1	2	3	4	5	1	2	3	4	5
Condition description		HEAT	SR:TET (1:1)	SR:TET (2:1)	Neat SR	TET (SR added to cartridge)	HEAT	SR:TET (1:1)	SR:TET (2:1)	Neat SR	TET (SR added to cartridge)
Final SR concentration (%)		0%	12.5%	16.6%	25.0%	50.0%	0%	12.5%	16.6%	25.0%	50.0%
CFU per 700 µL of sample buffer	0	0/20^*d*^ (0)	0/20 (0)	0/20 (0)	0/19 (0)	0/20 (0)	0/20 (0)	0/20 (0)	0/20 (0)	0/19 (0)	0/20 (0)
	3	6/20 (30.0)	6/19 (31.6)	8/18 (44.4)	5/19 (26.3)	5/20 (25.0)	3/20 (15.0)	2/19 (10.5)	5/18 (27.8)	3/19 (15.8)	1/20 (5.0)
	7	13/20 (65.0)	13/20 (65.0)	14/18 (77.8)	12/19 (63.2)	7/20 (35.0)	8/20 (40.0)	9/20 (45.0)	10/18 (55.6)	8/19 (42.1)	3/20 (15.0)
	28	16/25 (64.0)	24/25 (96.0)	22/25 (88.0)	22/25 (88.0)	10/24 (41.7)	7/25 (28.0)	15/25 (60.0)	16/25 (64.0)	13/25 (52.0)	1/24 (4.2)
	56	19/20 (95.0)	20/20 (100)	20/20 (100)	19/19 (100)	20/20 (100)	17/20 (85.0)	20/20 (100)	20/20 (100)	19/19 (100)	17/20 (85.0)
	112	18/19 (94.7)	*-*	*-*	*-*	20/20 (100)	18/19 (94.74)	*-*	*-*	*-*	18/20 (90.0)
	336	20/20 (100)	*-*	*-*	*-*	19/19 (100)	20/20 (100)	*-*	*-*	*-*	19/19 (100)
	Limit of detection (95% CI)	77.6 (51.2–104.0)	22.7 (14.2–31.2)	30.3 (19.9–40.7)	30.9 (21.5–40.3)	57.1 (42.4–71.7)	95.1 (68.9–121.2)	47.2 (35.1–59.2)	47.0 (33.7–60.2)	51.9 (38.5–65.2)	104.5 (80.7–128.3)
Errors (total errors/ total tests)		1^*e*^/145	0/104	1^*a*^/102	3^*a*^/104	2^*b*^^,*c*^/145	N/A	N/A	N/A	N/A	N/A

^*a*^ Error 2037: the cartridge integrity test failed at valve position 1390.

^*b*^ Error 2032: the ultrasonic horn could not be tuned properly.

^*c*^ Error 5007: [QC1] probe check failed.

^*d*^ Number of positive results/total number of samples tested (% positives); Positive test results were defined based on IS*6110*/IS*1081* Ct and *rpoB* Ct for Mtb and RIF, respectively.

^*e*^ Error 5007: [SPC] probe check failed.

^*f*^ CI, confidence interval; CFU, colony-forming unit; Ct, cycle threshold; *Mtb*, *Mycobacterium tuberculosis*; N/A, not applicable. RIF, rifampicin; SR, Xpert sample reagent; TET, Tris–EDTA–Tween buffer.

^*g*^ "-" refers to Not applicable.

### Performance of Xpert Ultra with sputum matrix versus tongue swabs

Given that tongue swabs processed with heating methods generate elevated pressures within the Xpert cartridge, which occasionally causes assays to be aborted as “errors” (8), we undertook an experiment to review the pressure in Xpert cartridges processed with the different swab conditions. However, we did not note errors of this type under any of our test conditions. Sputum samples will often produce high cartridge pressures and error rates when samples are not processed with SR. We, therefore, sought to assess the Xpert Ultra cartridge pressures obtained on processing sterile swabs dipped in TB-negative sputum with condition 1 and 3 protocols at 28 CFU in 700 µL of the relevant buffer.

Swabs containing a sputum matrix and 28 CFU *Mtb* showed a trend that was not statistically significant of higher in-cartridge pressures, with median in-cartridge pressures in tests of condition 1 versus condition 3 of 22.2 versus 17.9 psi, *P* = 0.11. (Fig. 4; Table S5). The Xpert Ultra tests of sputum matrix swabs detected *Mtb* in 79% of the samples in condition 1 versus 100% detection in condition 3 (*P* = 0.047) and detected RIF susceptibility in 47.4% of sputum matrix swabs in condition 1 versus 85% in condition 3 (*P* = 0.018) (Table 3). No false positive results for *Mtb* or RIF susceptibility were observed. Finally, the sputum swab matrix samples had higher mean IS*6110* Ct values in condition 1 versus condition 3 (25.22 versus 22.49, *P* < 0.001). No statistically significant differences were observed between swab matrix or processing method for *rpoB* Ct (Fig. 4B; Table S6) values, likely because many more RIF susceptibility assay results were negative in condition 1 and, thus, did not produce a Ct value.

**TABLE 3:** Xpert Ultra performance with *Mtb* spiked into buffer with swab samples processed using condition 1 (heat) versus condition 3 (diluted SR:TET 2:1) to test impact of sputum matrix and buffer type (TET vs PBS)^*a*^

Analyte		*Mtb* positivity, n/N (%) (based on IS*6110*/IS*1081* Ct)		RIF susceptibility, n/N (%) (based on *rpoB* Ct)	
CFU/700 µL	Conditions tested	Condition 1 (heat)	Condition 3 SR:TET (2:1)	Condition 1 (heat)	Condition 3 SR:TET (2:1)
28 CFU	Tongue swab	16/19 (84.2)	20/20 (100)	13/19 (68.4)	16/20 (80.0)
	Sputum swab	15/19 (79.0)	20/20 (100)	9/19 (47.4)	17/20 (85.0)
28 CFU	TET	N/A	15/15 (100)	N/A	13/15 (86.7)
	PBS	N/A	15/15 (100)	N/A	14/15 (93.3)

^*a*^ CFU, colony-forming unit; Ct, cycle threshold; *Mtb*, *Mycobacterium tuberculosis*; N/A, not applicable. PBS, phosphate-buffered saline; RIF, rifampicin; SR, Xpert sample reagent; TET, Tris–EDTA–Tween buffer.

### Performance of Xpert Ultra with phosphate-buffered saline versus Tris–EDTA–Tween buffer

We compared testing in our Tris–EDTA–Tween buffer versus testing in a PBS buffer, which might be preferred at many test sites, using *n* = 15 samples in each group. All steps for testing with PBS were the same as those for condition 3, except that the Tris–EDTA–Tween buffer was replaced with a PBS buffer. The Xpert Ultra performance was assessed at 28 CFU/700 µL. Detection was not different between the two buffers; Xpert Ultra detected *Mtb* in 100% samples with both PBS and Tris–EDTA–Tween and detected RIF susceptibility in 93.3% of PBS and 86.7% of the Tris–EDTA–Tween samples (*P* = 1) (Table 3; Table S7; Fig. 5).

## DISCUSSION

This study demonstrates the utility of several sample processing conditions using diluted Xpert SR to improve the sensitivity of Xpert Ultra to detect TB and RIF resistance in testing tongue swabs of people with presumed TB compared to the commonly used heat-based protocol. The 2:1 diluted SR buffer (condition 3) had a two-fold lower (i.e., improved) LOD compared to the commonly used heat-based inactivation method and, for biosafety reasons, was selected for further analysis. There was minimal influence of sputum matrix or dilution buffer on the performance of the Xpert Ultra assay using this condition.

Xpert Ultra testing of sputum for TB detection involves the addition of one volume of sputum to two volumes of Xpert SR (17). In a recent study by Andama et al. (8), processing tongue swabs with SR before Xpert Ultra testing has resulted in a sensitivity of 78% and a specificity of 100%, with sputum as the reference standard (8). The LOD of Xpert Ultra observed in the analytical component of Andama et al. with single- and double-swab SR methods and a final SR concentration of 66.6% (800 µL Tris–EDTA + 1,600 µL SR) was 101.7 and 76.5 CFU/swab, respectively. In comparison, the LOD of Xpert Ultra with boiling-based inactivation was 22.3 CFU/swab (8). We similarly observed that the odds of *Mtb* and *rpoB* detection were higher with the heat-based inactivation method than with 66.6% SR, although not significantly different. However, we tested sample processing conditions with a final SR concentration ranging from 12.5 to 50% to optimize a method as simple as Xpert Ultra’s sputum-based testing. We found that 16.6% of SR in our preferred condition 3 resulted in an LOD of 30.3 CFU per 700 µL sample, indicating that LOD can be improved using a lower concentration of SR. We chose condition 3 as our preferred condition for subsequent studies of sputum swabs and PBS buffer, even though condition 2, which contained 12.5% SR, had a lower LOD of 22.7. We made this selection because the two conditions had virtually identical numerical LODs for RIF susceptibility, and the higher concentration of SR has a known sterilizing activity against *Mtb* in sputum (21).

Our study also helped address concerns noted by previous studies on the role of tongue swab components in over-pressurization errors observed with Xpert Ultra testing using the boil inactivation method (19). We confirmed that in-cartridge pressure variations observed with heat-based processing in sputum matrix swabs could be minimized by treatment with diluted SR buffer. Our method may, therefore, enable improved sensitivity with reduced risk of over-pressurization errors.

Previous oral swab evaluations have used different types of sterile buffer matrices ranging from the Tris–EDTA buffer (8, 22), saline (11, 23), sterile lysis buffer (50 mM Tris pH 8.0, 50 mM EDTA, 50 mM sucrose, 100 mM NaCl, and 1% SDS) (12, 24, 25), to 7H9 + OADC + Tween-80 (26) for storage or dry swab sample processing (8, 9, 11). However, most analytical laboratory evaluations of Xpert Ultra or Xpert MTB/XDR have been performed with TET buffer as a standard test solution for sample processing (21, 27). Hence, we used this buffer to evaluate the LOD of tongue swabs with Xpert Ultra using the modified processing conditions in our study. However, many clinical laboratories in low-resource settings may have difficulty in procuring or making sterile TET buffer. We, therefore, evaluated the sensitivity of tongue swabs processed with the more readily available PBS. No difference in *Mtb* positivity or RIF susceptibility detection between condition 3 using TET buffer and PBS was observed. As our method also eliminates the need for additional laboratory equipment, the PBS-based condition may be of particular value in low-resource settings. It is likely that phosphate buffer without saline, as provided with sputum decontamination kits, would work just as well, and assessment of this in clinical specimens is underway.

The Cepheid proprietary sodium hydroxide- and isopropanol-containing SR was primarily designed to liquify and inactivate sputum samples. SR-inactivated *Mtb* cells are captured on a membrane filter in the Cepheid cartridge during automated sample processing in the Xpert cartridge (21). SR is known to be tuberculocidal (21, 28). Use of an SR-based sample processing method over the existing heat-based protocol, therefore, has the potential to reduce biohazard risk during tongue swab Xpert Ultra processing in a healthcare laboratory setting.

We used 0.05% Tween 80 in the 7H9 media used to make CFU dilutions. Tween 80 has been shown to reduce clumping of mycobacterial cells and is useful in preparing homogenous suspension of bacterial cells and has been used to make dilutions of Mtb cells in all of our analytic studies of Xpert assays (17, 27). Our test buffer also included Tween 20 as part of our TET swab elution buffer. Tween 20 may also have been acting as a dispersing method in our study, except where the swab was added directly to SR, which did not contain substantial amounts of Tween. However, no Tween formulations were present in the PBS and PB buffer comparisons, which showed a performance similar to the TET buffer; therefore, we do not think that Tween is an essential component for testing swabs using our recommended protocol, despite its theoretical benefit.

Although we have demonstrated a relatively simple procedure for tongue swab processing for Xpert Ultra testing, this study is limited by the lack of a clinical validation of our protocol. We thawed our *M. tuberculosis* cell stocks on ice. It is possible that the freeze thaw may have contributed to cell lysis, thereby improving the LOD of our assays. However, the same thawed stocks were used to make dilutions for all experimental conditions; therefore, we would not expect that this issue would affect the relative performance of each condition. Final LOD estimates will need to be assessed during clinical studies. It will be essential to study the utility of our processing methodology in tongue swabs collected from people with confirmed TB, smear-negative TB cases based on disease severity, and people with presumptive TB unable to produce sputum. Evaluation of sensitivity and specificity compared with other non-sputum sample types will also be important. In addition, specimens from various age groups and demographic subsets with different diets and lifestyles may impact the limit of detection and the error rate; therefore, our results will require confirmation in a future clinical study. However, the strength of our study is in its ability to analytically validate multiple sample processing methods in a systematic manner, which is very difficult to demonstrate in clinical studies.

In conclusion, the performance of Xpert Ultra for detection of *Mtb* positivity and RIF resistance in tongue swabs was improved using a brief incubation in an SR-containing buffer, followed by a 3:1 dilution of a non-SR-containing buffer when the treated sample is added into the Xpert cartridge, when compared with the existing heat-based protocol. This approach may increase the feasibility of using tongue swabs to screen for TB, thereby expanding the availability of TB testing to additional lower levels of the healthcare system and populations. Further studies are needed to assess the performance of this new tongue swab sample processing condition in a clinical setting.

## SUPPLEMENTAL MATERIAL

Tables S1 to S7 spectrum.02403-24-s0001.xlsx Supplemental tables provide raw data used to generate Fig. 2-5.

ASM does not own the copyrights to Supplemental Material that may be linked to, or accessed through, an article. The authors have granted ASM a non-exclusive, world-wide license to publish the Supplemental Material files. Please contact the corresponding author directly for reuse.
